# Hyperglycemia Induced by Chronic Restraint Stress in Mice Is Associated With Nucleus Tractus Solitarius Injury and Not Just the Direct Effect of Glucocorticoids

**DOI:** 10.3389/fnins.2018.00983

**Published:** 2018-12-19

**Authors:** Xiang Zheng, Wenjie Bi, Guizhi Yang, Jia Zhao, Jie Wang, Xiaojing Li, Xue Zhou

**Affiliations:** ^1^Department of Histology, Embryology and Neurobiology, West China School of Basic Medical Sciences & Forensic Medicine, Sichuan University, Chengdu, China; ^2^Department of Anatomy, Histology and Embryology, Chengdu Medical College, Chengdu, China; ^3^MOE Key Laboratory of Bioinformatics, Bioinformatics Division, Center for Synthetic and System Biology, Department of Automation, Tsinghua University, Beijing, China; ^4^Department of Histology and Embryology, Fuzhou Medical College, Nanchang University, Fuzhou, China

**Keywords:** chronic restraint stress, insulin-resistant hyperglycemia, neuron injury, apoptosis, dexamethasone, nucleus tractus solitarius, mouse

## Abstract

Chronic restraint stress (CRS) can affect hypothalamic-pituitary-adrenal (HPA) axis activity and increase glucocorticoid levels. Glucocorticoids are stress hormones that regulate multiple aspects of energy homeostasis. Stress also impairs glucose tolerance. The aim of this study was to investigate the cause of insulin-resistant hyperglycemia during CRS. We produced the CRS models (a 7-day restraint followed by a 3-day free moving procedure, total of 4 cycles for 40 days) in mice, detected the parameters related to glucose metabolism, and compared them to those of the dexamethasone (DEX) injection (0.2 mg/kg i.p., also a 4 cycle procedure as the CRS). The results showed that the CRS induced a moderate (not higher than 11 mmol/L) and irreversible insulin-resistant hyperglycemia in about 1/3 of the individuals, and all the restrained mice had adrenal hypertrophy. CRS induced the apoptosis of neurons in the anterior part of commissural subnucleus of nucleus tractus solitarius (acNTS) in the hyperglycemic mice, and acNTS mechanical damage also led to insulin-resistant hyperglycemia. In contrast, in the DEX-treated mice, adrenal gland atrophy was evident. The glucose and insulin tolerance varied with the delay of determination. DEX exposure *in vivo* does not induce the apoptosis of neurons in NTS. This study indicates that restraint stress and DEX induce metabolic disorders through different mechanisms. During CRS, injury (apoptosis) of glucose-sensitive acNTS neurons cause dysregulation of blood glucose. This study also suggests the mouse restraint stress model has value as a potential application in the study of stress-induced hyperglycemia.

## Introduction

Numerous studies have confirmed that stress can lead to abnormal body function and even cause disease (De Kloet et al., 2005; Feng et al., 2012; Hackett and Steptoe, 2017; Ménard et al., 2017; Kivimäki and Steptoe, 2018). Hypothalamic-pituitary-adrenal (HPA) axis is activated under stress. In chronic stress, high levels of glucocorticoids lead to hypertension, insulin resistance, and hyperlipidemia (Rafacho et al., 2014). Because glucocorticoids can cause insulin resistance, investigators have used glucocorticoid injection, or oral administration, to create experimental animal models of insulin-resistant hyperglycemia (Rafacho et al., 2014;We have changed “Spencer, 2017” as “Spencer and Deak, 2017” inside the text as per the reference list. Kindly confirm if this is fine. Spencer and Deak, 2017). Synthetic glucocorticoid – dexamethasone (DEX) has a long half-life, strong efficacy, and is commonly used in chronic experiments. DEX reduces the efficiency of insulin signaling sensitive glucose transporters, reduces peripheral tissue glucose uptake, and increases hepatic glucose output (Pasieka and Rafacho, 2016), thereby increasing blood glucose. We observed that chronic restraint stress (CRS) can lead to insulin resistance and hyperglycemia in no more than 50% rat (Liang et al., 2013). Mice exposed to chronic variable stress also showed decreased insulin sensitivity (Jelenik et al., 2018). So, is stress-induced hyperglycemia a direct effect of glucocorticoids? In previous experiments, the results obtained by direct administration of DEX were different (e.g., body weight and glucose tolerance) from those of CRS models. Therefore, it is supposed that in addition to the role of hormones, neuromodulation is also a factor in the maintenance of the steady state of glucose metabolism. Changes in brain neurotransmitter synthesis in CRS rats (Li et al., 2012) have been observed. It was also reported that chronic stress can cause increased excitability in the solitary nucleus, posterior hypothalamus, and some limbic structures in the brain (Flak et al., 2012). It has not been reported if chronic stress damages neurons in these brain regions. Thus, further studies should be conducted to determine if damage to these neurons affects glucose metabolism. Recent studies have revealed that chronic psychological stress (De Fronzo et al., 2015; Hackett and Steptoe, 2017) and lack of physical exercise (Vaynman and Gomez-Pinilla, 2006) are risk factors for disorders of glucose metabolism. However, current animal models of glucose metabolism disorders have not fully considered the factors of stress-induced neurological changes. The restraint model is generally used to study the mechanism of stress-induced learning and memory impairment, which simultaneously simulates psychological stress and exercise limitation. In this study, mice were used to compare the main differences between the CRS model and the chronic DEX injection model, focusing on the phenomenon of apoptotic injury of the glucose sensitive neurons in the nucleus tractus solitarius (NTS) in the medulla oblongata. The purpose of the study is to demonstrate that stress-induced brain neuronal injury can be a cause of abnormal glucose metabolism. Results showed that CRS and DEX chronically administered mice showed substantial differences in body weight gain, glucose metabolism, adrenal cortical changes, and serum hormone levels. CRS, but not DEX, caused apoptotic injury in the glucose-sensitive neurons located in the medulla oblongata of mice, which developed insulin-resistant hyperglycemia. Direct mechanical damage to this area also caused insulin-resistant hyperglycemia and exhibited hormone level changes similar to those of CRS.

## Materials and Methods

### Animals

Eight weeks old male KM mice (closed colony with heterozygous genetic background; body weight 28.6 ± 1.21 g, fasting blood glucose level was 5.4 ± 0.3 mmol/L) were purchased from Animal Experiment Center of Sichuan University. Experimental procedures were approved by the ethics committee for laboratory animals at Sichuan University and in strict accordance with the National Institutes of Health Guide for the Care and Use of Laboratory Animals (publication no. 85-23, revised 1985). All mice were maintained under a 12 h light/dark cycle with free access to food and water. Room temperature was 22 ± 2°C, with humidity of 60 ± 5%. The size of cages is 290 mm × 180 mm × 150 mm, and the density is 5 mice per cage.

### CRS Modeling

**FIGURE 1:**
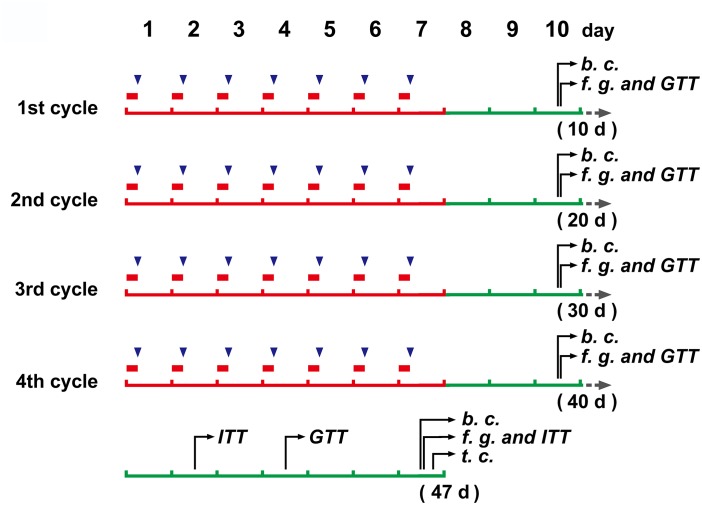
The schedule of main operations and detections of the study. 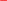 (in red color), a 6 h restraint; 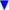, DEX injection (to mice without restraint); b. c., blood collection; f. g., determination of blood glucose levels after fasting; GTT, glucose tolerance test; ITT, insulin tolerance test; t.c., tissue collection.

Mice (*N* = 20) were placed in restraint devices (referred to the reported studies of Bowers et al., 2008; Guo et al., 2017, with a few modification. The detailed operation procedure see the “Mouse restraint operation” in Supplementary Material) and restrained individually for 6 h every day from 0:00 to 6:00 am at 16–18°C. The mice continued to restrain for 7 days and then had a three day off. Total of 4 cycles were performed (Figure 1). Control mice (*N* = 8) and CRS mice entered the restraint devices at the same time, but then the control mice were released and free to move.

### DEX Injection

After body weight measurement (with an electronic balance, JA31002), mice (*N* = 8) were injected intraperitoneally with 0.2 mg/kg (4 ml/kg with a concentration of 2.5 mg/50 ml) of DEX (Sigma-Aldrich, D1756) at 6:00 a.m. every day, which was dissolved in the solvent made of 10% ethanol, 30% propylene glycol and 60% phosphate buffered saline on the day of injection (This formulation refers to the report of Barnum et al., 2008, and makes some adjustments for DEX. The concentration of DEX is determined by preliminary experiments. See Supplementary Figure S1). The DEX injection also followed the 7 days on + 3 days off cycle like the CRS modeling for a total of 4 cycles (Figure 1). Control mice (*N* = 8) were injected intraperitoneally with the same volume of solvent as DEX, and the injection time and cycle were the same as those of DEX injection.

### Glucose Solution Injection

Untreated mice (*N* = 3) were fasted for 6 h and then did IP injection with a saline solution of 30% glucose at a dose of 2 g/kg. The control group (*N* = 3) injected the same volume of saline. After 1.5 h, the brain tissues were collected for detection of c-Fos positive neurons associated with elevated blood glucose (see below).

### Glucose and Insulin Tolerance Tests (GTT and ITT)

Blood glucose levels of each mouse were monitored at the end of each cycle by a portable glucose meter (Lifescan, OneTouch ultra). The mice were fasted for 6 h, the tail tip was cut and the first drop of blood was discarded, and then the blood glucose concentration was measured. In the GTT, mice were injected with glucose solution (i.p., 2 g/kg), and glucose levels were monitored at the 30, 60, 90, and 120 min. In the ITT (2–3 days after the GTT, only twice in this study, see Figure 1), animals were intraperitoneally challenged with 0.75 mIU/g of human insulin (Novolin R, Novo Nordisk, Denmark). The glucose detecting procedure was the same as described above.

### Serum Hormone Determination

The tail tips of the mice were cut, and the blood was collected by gently squeezing the tail. The blood allowed to clot at room temperature for 30 min, and centrifuged at 4°C, 1000 g for 20 min. Take the supernatant for testing (approximately 20 μl of serum was needed for a single test). The serum samples for corticosterone determination were treated for an additional 1 h in a 75°C water bath (to denature the corticosteroid binding globin). Corticosterone and insulin concentrations were determined following the double antibody sandwich (for insulin, Cloud-Clone, L151228778) and competitive inhibition (for corticosterone, Cloud-Clone, L160105155) ELISA protocol, respectively. The data were determined by measuring the absorbance at 450 nm using a microplate reader (PERLONG DNM-9602).

### Mechanical Damage of the NTS

Mice (*N* = 6) were anesthetized with ketamine/xylazine (ketamine: 60 mg/kg; xylazine: 10 mg/kg). After the hair was removed, the skin was incised to expose the posterior portion of the skull. The mice were bound in the prone position and the needle points were accurately marked on the brain stereotaxic apparatus. The vertical insertion of needle at the coronal plane interaural -3.76 mm, bregma -7.56 mm (referred to the brain stereotaxic atlas, Paxinos and Franklin, 2001) and 0.2 mm away from midline (only on the right side) can damage the anterior region of the commissural nucleus of NTS. We drilled the skull with the needle of a 5 ml syringe, then used a 1 μl microsyringe needle for vertical puncture to a depth of 4.5 mm, keeping the needle still for 10 s before retracting. The puncture depth of sham surgery (*N* = 6) was 3.6 mm to ensure that the needle did not enter the medulla. The scalp were conventionally sutured. Mice naturally wake up under warm conditions. The fasting blood glucose level was tested on the 2nd, 5th, 7th, and 10th days after operation. The GTT and ITT were performed on the 7th and 10th day, respectively, after operation. Tissue samples were collected on the 12th day.

### Tissue Fixation, Histology, Immunohistochemistry and TUNEL Assay

After weighing, mice were anesthetized with an overdose of sodium pentobarbital and perfused intracardially with 4% neutral buffered paraformaldehyde. Brain, adrenal glands and pancreas were removed, weighed with electronic balance and post-fixed in the same fixative overnight at 4°C. Specimens were routinely embedded in paraffin and serially sectioned at 6 μm thickness for HE staining, TUNEL assay (brain) and immunohistochemical staining. The method for islet volume measurement was referred to the literature (Rafacho et al., 2009) and adjusted according to the characteristics of the mouse (6 islets of each mouse were randomly selected from serial HE-stained sections of the pancreas, and the volume was calculated using the Cavalieri principle). Immunohistochemistry was performed followed the SABC procedure (SPN-9001, ZSGB-BIO). Primary antibodies against insulin, rabbit polyclonal IgG, 1:150 (bs-0056R, Bioss), c-Fos, rabbit polyclonal IgG, 1:120 (ab209794, Abcam) and Caspase-3 rabbit polyclonal IgG, 1:100 (bs-0081R, Bioss) were used for immunohistochemistry staining. The result of the reaction was visualized by the DAB method. TUNEL assay was conducted following the commercial kit’s instructions (Beyotime C1091, DAB method).

### Statistical Analysis

Results were expressed as means ± SEM. Normal distribution and equality of variances were examined using Shapiro–Wilk test and Levene’s test. Body weight gain, corticosterone level, GTT and ITT data were compared using repeated measures two-way ANOVA, with time as the variable factor, and treatment as fixed factors. If ANOVA revealed a significant difference, a *post hoc* Bonferroni test was used to further characterize the group differences. Glucose and insulin levels at a single time-point, adrenal indices and islet volumes were compared using one-way ANOVA, and a *post hoc* Student–Newman–Keul’s test was used to further characterize the group. Data between groups of acNTS injury and the sham operation were compared with independent Student’s *t*-tests. Statistical analyses were performed using IBM SPSS (version 22). *P* < 0.01 was considered significant.

## Results

### Body Weight, Fasting Blood Glucose and Insulin Levels

**FIGURE 2:**
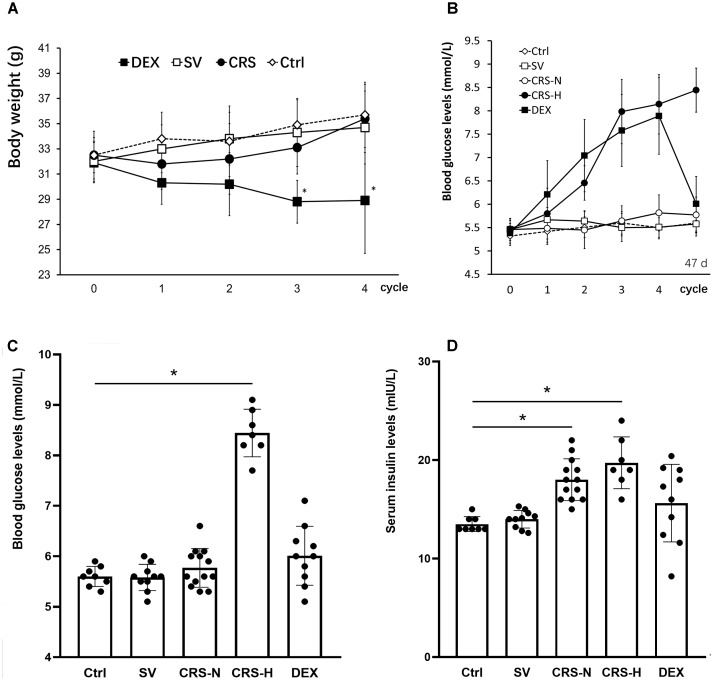
Comparison of body weight changes, fasting blood glucose and serum insulin levels. **(A)** The effect of restraint on weight gain is transient, while DEX significantly reduces body weight in mice. **(B)** From the 3rd cycle of CRS, fasting blood glucose levels differentiated, and fasting blood glucose levels in 7 of the 20 mice increased significantly and remained high thereafter. Fasting blood glucose in DEX-administered mice showed a trend of increasing gradually. The fasting blood glucose levels in **(B)** were detected at the end of each restraint cycle. **(C)** Fasting blood glucose levels on the 7th day after the end of the 4th cycle. At this time, fasting blood glucose in 7 CRS-hyperglycemia mice was significantly higher than that in the non-restraint control, and there was no significant difference in blood glucose levels in the other groups. **(D)** Fasting serum insulin levels on the 7th day after the end of the 4th cycle. Serum insulin levels in CRS mice are significantly higher than those of controls regardless of hyperglycemia. The individual differences in serum insulin levels in DEX injected mice were large, and overall there was no significant difference from mice injected with solvent. Ctrl, normal control; SV, solvent injection; CRS-N, CRS-normoglycemia; CRS-H, CRS-hyperglycemia. Data in **(A,B)** were shown as means ± SEM; ^∗^*p* < 0.01.

Chronic restraint stress mice exhibited a decrease in body weight at the end of the 1st cycle and recovered weight gain in the following cycles. By the end of the 4th cycle, there was no significant difference compared to the non-restraint control. The weight of DEX injected mice slowly decreased, showing a significant difference between the solvent injected mice from the 3rd cycle [There was a significant main treatment (group) effect, *F* = 9.564, *P* < 0.01; *post hoc* analysis showed a weight decrease of the DEX group, *P* < 0.01]. There was no significant difference in body weight between solvent injected and non-restraint control mice. The results are shown in Figure 2A.

Seven of the CRS mice exhibited a trend of increasing fasting blood glucose. Fasting blood glucose levels increased from the 2nd cycle and were significantly higher than the others at the end of the 4th cycle (Figure 2B). Fasting blood glucose levels in DEX injected mice continue to increase (Figure 2B). On the 7th day after the end of the 4th cycle, the 7 CRS mice still maintained fasting hyperglycemia, and there was no significant difference in the other individuals [There was a significant difference among groups, *F* = 9.642, *P* < 0.01, and *post hoc* analysis showed an elevated glucose level of the CRS-hyperglycemia group (*P* < 0.01); Figure 2C]. Serum insulin levels were also significantly elevated in these 7 hyperglycemic mice (*F* = 5.614, *P* < 0.01, and *post hoc* analysis showed that both the CRS-hyperglycemia and the CRS-normoglycemia group were significantly different from the control, *P* < 0.01; Figure 2D), suggesting insulin resistance.

### Variations in Glucose and Insulin Tolerance

**FIGURE 3:**
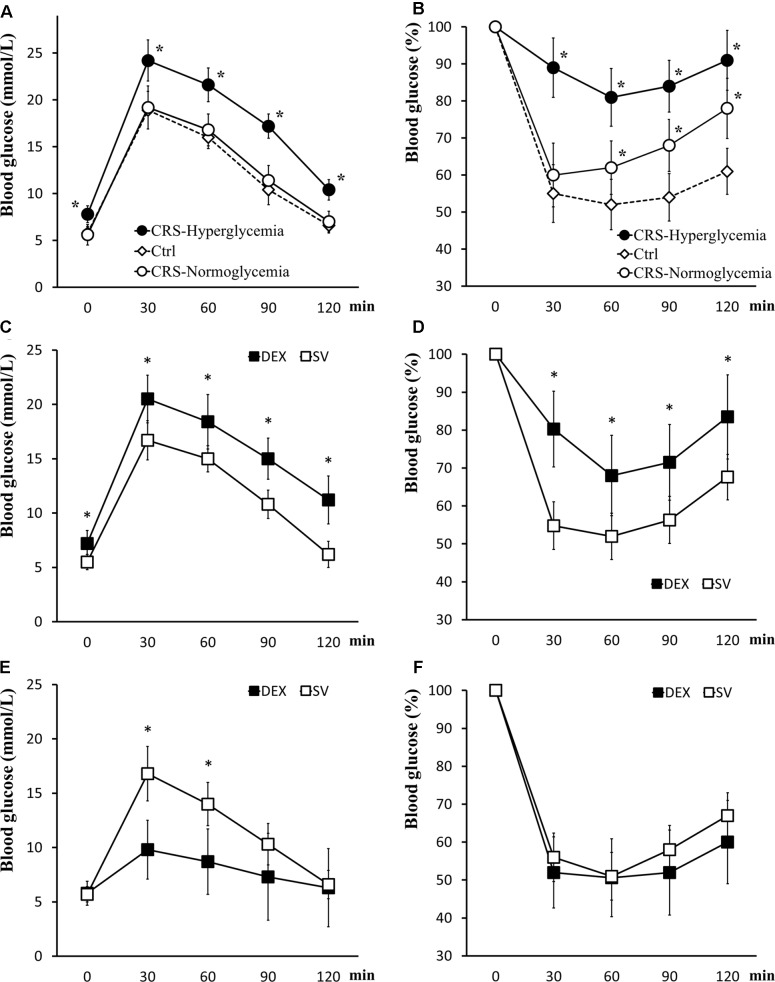
Glucose tolerance and insulin tolerance tests. **(A,B)** GTT and ITT in CRS and control mice. Both CRS-hyperglycemic and CRS-normoglycemic mice showed insulin resistance, but the latter had normal glucose tolerance. **(C,D)** Comparison of glucose tolerance and insulin tolerance in DEX and solvent (SV) injected mice 3 days after DEX withdrawal. At this time, DEX injected mice showed insulin-resistant hyperglycemia. **(E,F)** 7 days after the withdrawal of DEX, the efficiency of glucose uptake in DEX injected mice was increased and no longer showed fasting hyperglycemia and insulin resistance. Data were shown as means ± SEM, ^∗^*p* < 0.01.

Mice that exhibited fasting hyperglycemia after CRS showed significant insulin resistance in tolerance tests (for GTT, there was a significant main effect of groups, *F* = 50.894, *P* < 0.01; *post hoc* analysis revealed that the CRS-hyperglycemia group was significantly different from either the CRS-normoglycemia or the control group, *P* < 0.01), suggested decreased glucose uptake efficiency. In mice with normal fasting glucose level after CRS, glucose tolerance was normal but insulin sensitivity decreased (for ITT, data showed a significant main effect of groups, *F* = 62.642, *P* < 0.01; *post hoc* analysis revealed that both the CRS-hyperglycemia and the CRS-normoglycemia group were different from the control, *P* < 0.01. Figures 3A,B). The hyperglycemic state of CRS mice continues for at least 3 weeks after the end of the restraint (Supplementary Figure S2). Chronic DEX injected mice showed insulin-resistant hyperglycemia compared with the solvent injected mice until days 3 – 5 after the end of injection (for GTT, there was a significant main effect of groups, *F* = 36.882, *P* < 0.01, and for ITT, *F* = 26.768, *P* < 0.01; Figures 3C,D). 7–10 days after the end of chronic DEX injection, glucose utilization and insulin sensitivity are increased instead of insulin resistance (for GTT, there was a significant main effect of groups, *F* = 16.276, *P* < 0.01, and for ITT, *F* = 4.568, *P* > 0.01; Figures 3E,F).

### Adrenal Index, Adrenal Morphology and Serum Corticosterone

**FIGURE 4:**
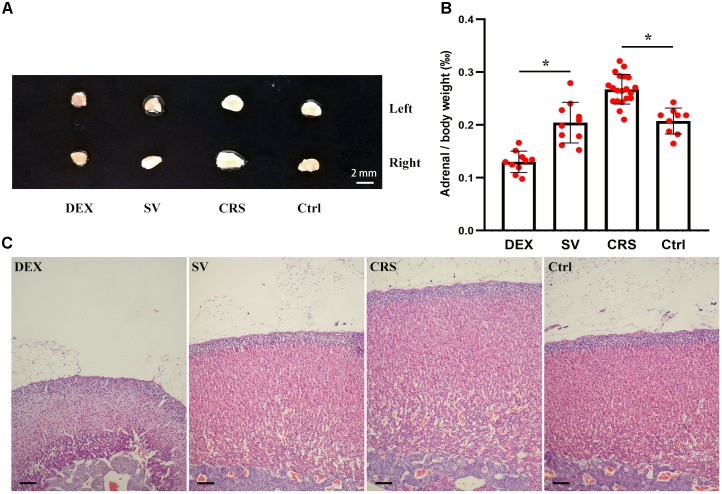
Effects of DEX injection and CRS on adrenal weight and cortical morphology. **(A)** The adrenal size of DEX injected mice was smaller than that of solvent (SV) injected mice, whereas the adrenal glands of CRS mice were larger than the non-restraint control (Ctrl) group. **(B)** Comparison of the adrenal mass to body weight ratio (adrenal index). The data showed that the adrenal index of mice injected with DEX was significantly lower than that of mice injected with solvent. The adrenal index of CRS mice was significantly higher than that of the control group. Data were shown as means ± SEM, ^∗^ *p* < 0.01. **(C)** The HE staining sections of mouse adrenal cortex. The CRS mice had hyperplasic adrenal cortex (about 68 cell layers or 1,000 μm in thick, compared to 54 cell layers or 750 μm for the normal control). On the contrary, the adrenal cortex of DEX injected mice was only about 37 cell layers or 500 μm in thick, much thinner than the solvent injected mice. Scale bar = 100 μm.

The effect of CRS and DEX chronic injection on the adrenal gland is different. The bilateral adrenal glands in CRS mice were enlarged (Figure 4A), and the adrenal index (adrenal mass/body weight) was elevated (*F* = 13.625, and *post hoc* analysis revealed a significance for the CRS compared to the control group, *P* < 0.01; Figure 4B). The cortical thickness was increased while the microstructure was normal (Figure 4C). In chronic DEX injected mice, there were adrenal atrophy, reduced adrenal index (*post hoc* analysis revealed a significant between the DEX and SV group, *P* < 0.01), and reduction in thickness of the zona fasciculata (Figures 4A–C).

**FIGURE 5:**
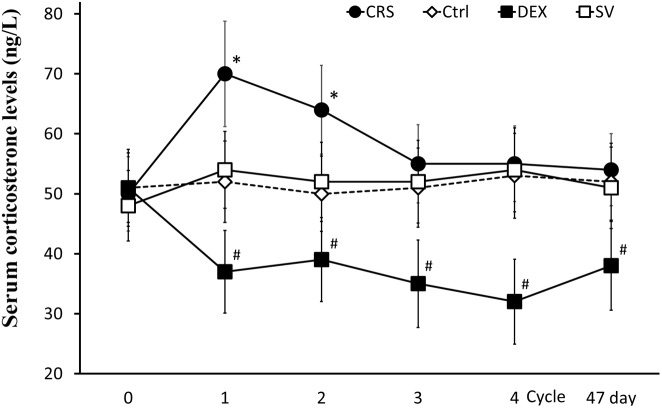
Changes in serum corticosterone levels. Serum corticosterone levels in CRS mice increased during the first two cycles (regardless of whether they developed hyperglycemia) and returned to normal after the 3rd cycle. Serum corticosterone remained low in DEX injected mice. There was no significant difference between solvent injection (SV) and normal controls (Ctrl). Data were shown as means ± SEM; ^∗^ compared with control mice, *p* < 0.01; ^#^ compared with solvent injection mice, *p* < 0.01.

At the end of the 1st cycle of CRS, serum corticosterone levels increased significantly and then fell back. After the 3rd cycle, there was no significant difference compared to non-restraint control mice (there was a significant main effect of groups, *F* = 16.334, *P* < 0.01; *post hoc* analysis revealed a significant difference between the CRS and the control group of the 1st and 2nd cycles, *P* < 0.01; Figure 5). The corticosterone level elevated by the restraint stress recovered within 2–3 h (Supplementary Figure S3). Therefore, the data obtained after 3 days of rest at the end of each cycle is not affected by operational stress. The serum corticosterone levels in DEX injected mice were consistently lower than those in solvent injected mice (*post hoc* analysis showed that the corticosterone level of DEX group was significantly different from that of the SV group, *P* < 0.01; Figure 5).

### Islet Morphology, Volume and Insulin Immunohistochemical Observation

**FIGURE 6:**
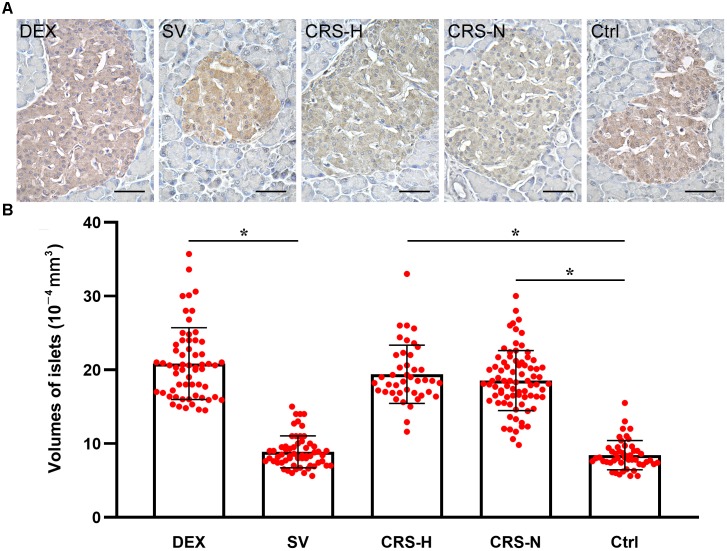
Insulin immunoreactivity and average volume of islets. **(A)** Insulin immunohistochemical staining. The islets of CRS-hyperglycemic and CRS-normoglycemic mice had reduced insulin immunoreactivity. The histological structure of islets in each group did not change. Bar = 50 μm. **(B)** Comparison of average volume of islets. Both DEX injection and CRS can increase islet volume. Islets of both CRS-hyperglycemic and CRS-normoglycemic mice were enlarged. SV, solvent injection; CRS-H, CRS-hyperglycemia; CRS-N, CRS-normoglycemia; Ctrl, normal control. Data in **(B)** were shown as means ± SEM, ^∗^*p* < 0.01.

Compared with the non-restraint control, there was no change in the microscopic structure of islets in CRS mice. The insulin immunoreactivity in islets was decreased, and the average islet volume was significantly increased (*F* = 11.246, *P* < 0.01; *post hoc* analysis revealed that both CRS-hyperglycemia and CRS-normoglycemia group were significantly different from the control, *P* < 0.01). There was no correlation between this effect and the occurrence of fasting hyperglycemia (Figures 6A,B). Islet morphology in DEX injected mice did not change, but insulin immunoreactivity and the average islet volume increased compared to the solvent injected mice (*post hoc* analysis showed a significant difference between the DEX and SV group, *P* < 0.01; Figures 6A,B).

### Morphology and Histochemical Staining of NTS

**FIGURE 7:**
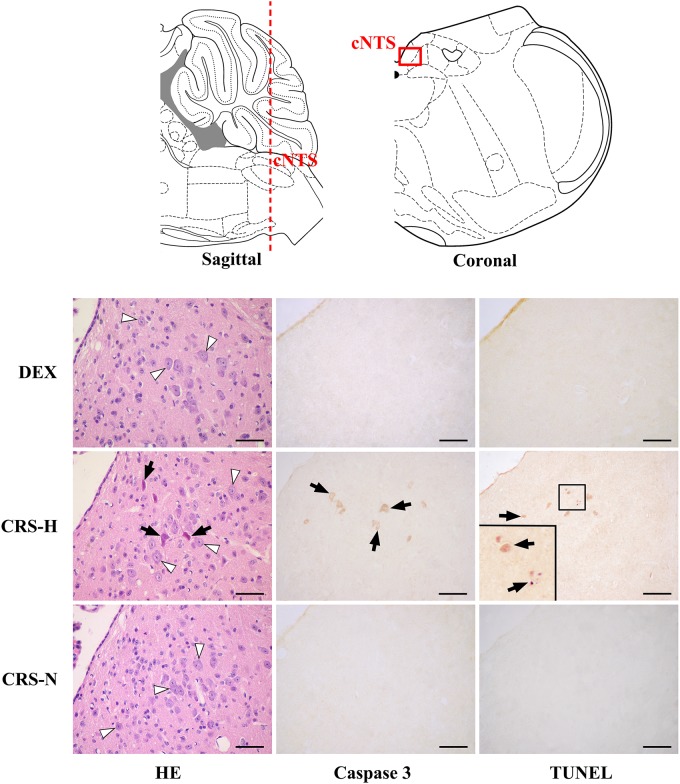
Neuronal apoptosis in acNTS of CRS mice. The coronal section was interaural –3.76 mm, bregma –7.56 mm. CRS-H, CRS-hyperglycemia; CRS-N, CRS-normoglycemia; Δ Normal neurons; ↑ Apoptotic neurons. Bar = 100 μm.

Caspase-3 positive cells and TUNEL positive staining were found in multiple regions of CRS hyperglycemic mice, suggesting apoptotic injury (Table 1). Among them, anterior part of the commissural subnucleus of NTS (acNTS) had neuronal injury after CRS and is excited by elevated blood glucose (Supplementary Figure S4). In the acNTS of CRS hyperglycemic mice, the nucleus and cytoplasm of a part of the neurons showed concentrated staining. Caspase-3 and TUNEL positive neurons were present in this area. No positive staining was observed in both CRS-normoglycemic and DEX injected mice (Figure 7). This apoptotic injury in acNTS was also observed at the end of the 1st restraint cycle (Supplementary Figure S5). Therefore, CRS can cause glucose-sensitive neurons apoptosis in NTS, while DEX does not have this effect.

**Table 1:** Neuronal apoptosis after CRS and glucose sensitive brain regions.

Caspase-3 expression and TUNEL positive staining regions (CRS-hyperglycemic mice compared to the CRS-normoglycemic mice)	Whether it can be excited by glucose^#^
Prefrontal cortex (mainly in medial prefrontal cortex)	No
Hippocampal structure	No
acNTS	Yes^##^
pcNTS	No
Cerebellar cortex (variable regions)	No


*Structural names refer to the mouse brain stereotaxic atlas (Paxinos and Franklin, 2001). acNTS, Anterior part of the commissural sub-nucleus of NTS; pcNTS, Posterior part of the commissural sub-nucleus of NTS. ^#^Compared with saline injection, the c-Fos positive nuclei increased significantly after glucose injection. ^##^See Supplementary Figure S4 in Supplementary Material*.

### Effect of acNTS Mechanical Injury on Blood Glucose Regulation in Mice

**FIGURE 8:**
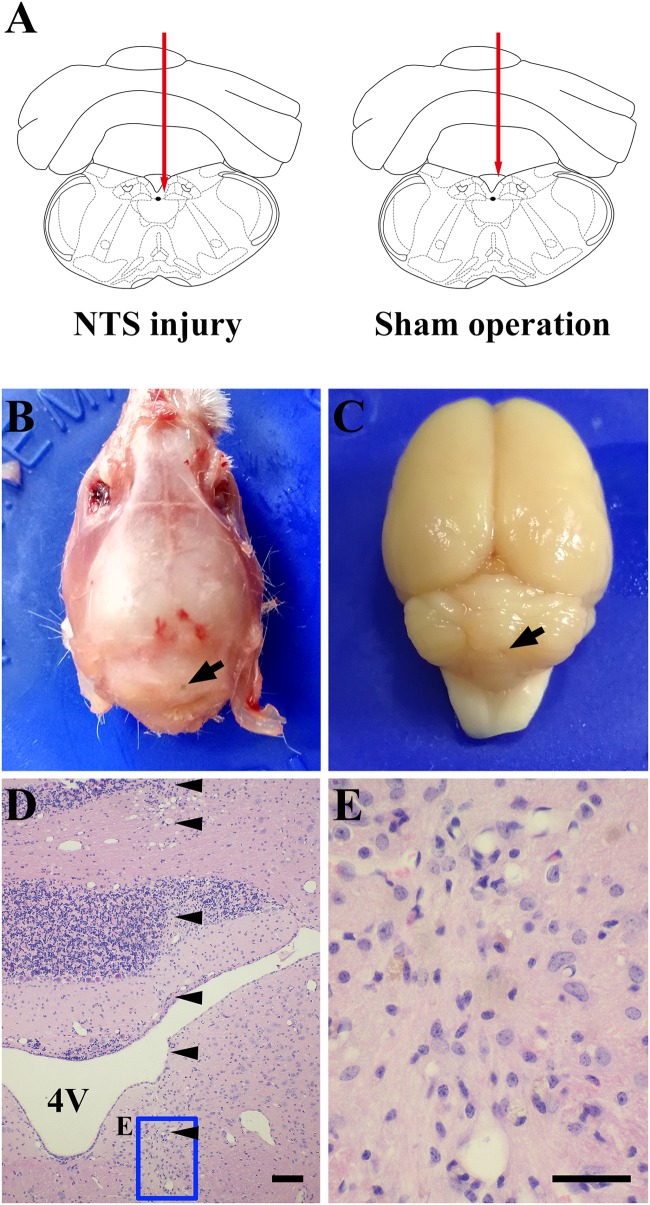
One case shows that acNTS mechanical damage which leads to high blood sugar. The time of tissue collection was the 12th day after surgery. **(A)** Needle route in the coronal section interaural –3.76 mm, bregma –7.56 mm. **(B)** The arrow indicates the position of the needle point on the dorsal side of the skull. **(C)** The arrow indicates the location of the needle insertion point on the dorsal surface of the cerebellum after removal of the skull. **(D)** HE staining low magnification imaging. 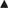 Indicates the needle route, bar = 200 μm. **(E)** HE staining high magnification image for the end area of the needle, bar = 100 μm.

**FIGURE 9:**
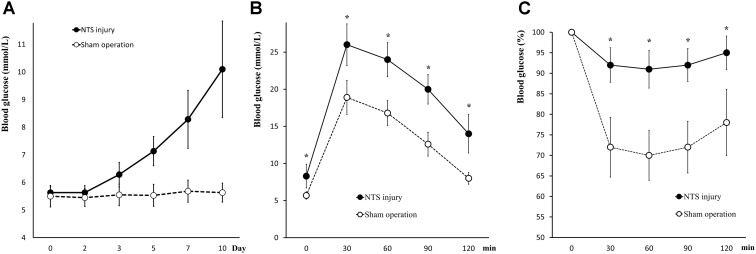
Changes in glucose metabolism in acNTS injured mice. **(A)** The trend of postoperative fasting blood glucose levels. Mechanical damage to acNTS can cause fasting blood glucose to increase gradually. **(B)** GTT. **(C)** ITT. Results showed that acNTS injured mice exhibit insulin-resistant hyperglycemia. Data in **(B,C)** were shown as means ± SEM, ^∗^*p* < 0.01.

Positioning acupuncture can cause mechanical damage of approximately 200 μm in diameter in mouse acNTS (Figures 8A–E). After injury, fasting blood glucose levels were in the normal range at beginning, and then gradually increased. Five days after surgery, fasting blood glucose levels were higher than those of sham-operated mice and continued to increase (Figure 9A). GTT and ITT showed that NTS injury leads to insulin resistance hyperglycemia (for GTT, there was a significant main effect of groups, *F* = 64.354, *P* < 0.01, and for ITT, *F* = 62.216, *P* < 0.01; Figures 9B,C) and NTS injured mice are less efficient in using blood glucose.

**FIGURE 10:**
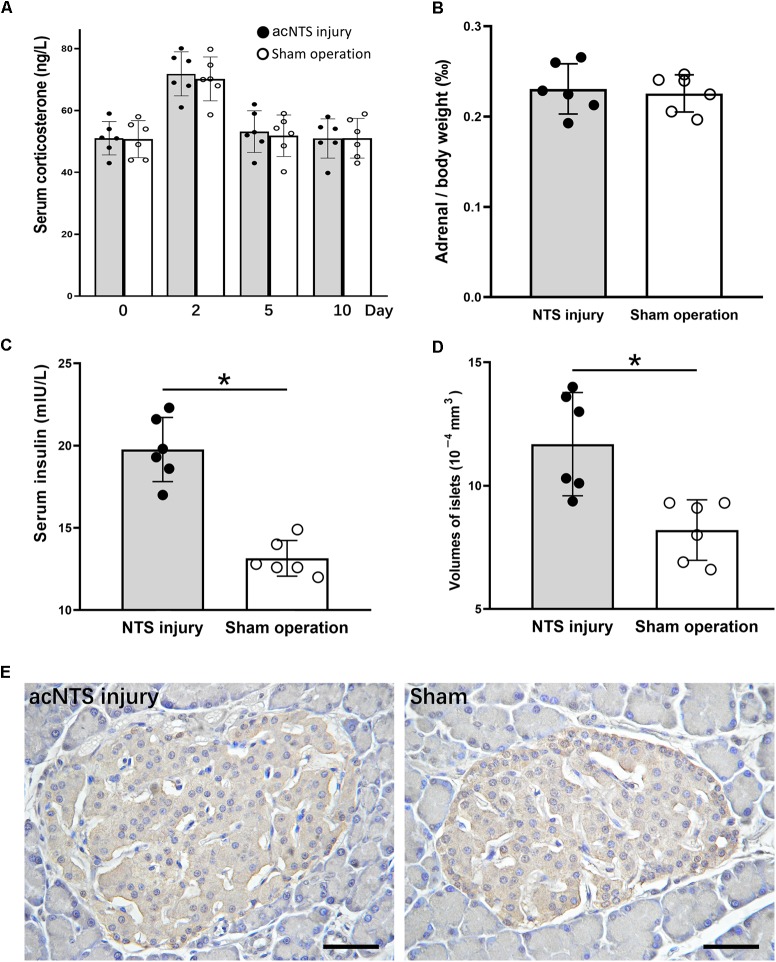
Changes in adrenal glands and pancreas in acNTS injured mice. **(A)** Changes in serum corticosterone levels. Two days after surgery, both surgical and sham mice showed a transient increase in corticosterone levels. **(B)** Adrenal index (the ratio of adrenal mass to body weight) did not change after acNTS injury. **(C)** Serum insulin levels in acNTS injured mice are significantly elevated. **(D)** The average volume of islets of acNTS injured mice was significantly increased. **(E)** Insulin immunohistochemical staining, scale bar = 100 μm. Data in **(B–D)** were shown as means ± SEM, ^∗^*p* < 0.01.

Serum corticosterone levels in both acNTS-injured and sham-operated mice increased in the first 2 days after injury. There was no statistical difference between the two groups, suggesting a surgically induced transient stress response. After this, serum corticosterone fell back to the same level as before surgery (Figure 10A). There is no statistical difference in adrenal index between acNTS injury and sham operation (*P* > 0.05; Figure 10B). After acNTS injury, serum insulin levels were significantly higher than in sham-operated groups (*P* < 0.01), and the mean islet volume increased (*P* < 0.01; Figures 10C,D). Considering that there was no significant difference in the intensity of insulin immunostaining between the injury and sham groups (Figure 10E), this result suggested that insulin synthesis and secretion were enhanced.

## Discussion

Our previous study established a rodent CRS model that can replicate stress hyperglycemia in 32.5% of the rat (Liang et al., 2013) and present brain changes consistent with elevated blood glucose (Li et al., 2012). How CRS causes insulin-resistant hyperglycemia is unclear. Studies indicated that the HPA axis is activated after chronic stress (Pacák and Palkovits, 2001; Fulford and Harbuz, 2005; McEwen, 2008). After activation of the HPA axis, glucocorticoid secretion increased. Glucocorticoids in the blood cause a decrease in the efficiency of insulin receptor signaling pathways in peripheral tissues (De Guia et al., 2014), resulting in insulin resistance. Glucocorticoids also act on the brain and exert a damaging effect by promoting neuroinflammatory reactions and neuronal apoptosis (Frank et al., 2012; Zhang et al., 2017). This study directly injected the long-acting synthetic glucocorticoid DEX according to the same operating cycle as the CRS. The comparison between the CRS model and the DEX chronic administration model showed obvious differences between the two models in many aspects, reflecting the difference in the pathways that cause changes in blood glucose metabolism.

Chronic restraint stress caused persistent insulin-resistant hyperglycemia in about 1/3 of mice. Injury of neurons (our results suggest apoptosis) was observed in NTS of CRS-hyperglycemic mice. The neurons of acNTS are excited after the injection of glucose solution. Therefore, after CRS-induced apoptosis of these neurons, blood glucose regulation was affected. The acNTS was mechanically damaged by acupuncture and the fasting blood glucose of mice gradually increased. These results demonstrate that NTS is a structure that is impaired by CRS and is clearly related to the function of blood glucose metabolism. DEX administration did not cause apoptosis in NTS neurons. CRS can also cause apoptosis in some cerebral cortical neurons, mostly at the medial prefrontal cortex, but the cerebral cortex is not directly excited by glucose. After acNTS injury, serum insulin levels and islet volume increased in mice, and these changes were consistent with changes induced by CRS. However, acNTS mechanical injury did not cause adrenocortical hyperplasia, and serum corticosterone levels did not change significantly except for a transient increase of 2 days postoperatively (sham increased synchronously). That is to say, HPA was activated during a certain period of CRS, and pure acNTS injury did not have a lasting effect on the HPA axis.

Unlike CRS, DEX administration has the same effect on all subjects. In order to induce insulin-resistant hyperglycemia, the concentration of DEX that should be given and how long it is administered has been reported with different results (Rafacho et al., 2014). After comparison, we believe that for chronic experiments, a dose of 0.2 mg/kg can exert its effect without causing excessive weight loss, nutrient deficiency, or other cachexia changes (Supplementary Figure S1). At no more than 5 days after the end of DEX administration, insulin-resistant hyperglycemia was present. However, when measured again after 7 days of recovery, glucose utilization and insulin sensitivity was reversed. Glucocorticoids can cause insulin secretion (not synthesis) to be blocked (Ferris et al., 2005). If hormone resistance occurs during chronic administration, it may cause hyperinsulinemia. Most of the experiments reported in the literature were carried out 1–3 days after the hormone administration, and little attention was paid to the results after sufficient recovery. Some researchers have observed the reversal of this glycemic effect (Rafacho et al., 2010; Fransson et al., 2013). As in this study, we also observed a significant increase in insulin synthesis in mice chronically injected with DEX. The reason may be that after the withdrawal of DEX, on the one hand, insulin secretion increases, and on the other hand, the ability of cells in the body to take up glucose rebounds. Therefore, the target cells were more sensitive to insulin. Therefore, the effect of DEX on blood glucose metabolism is quick but temporary, whereas CRS causes persistent insulin resistance, suggesting CRS induced hyperglycemia, which persists for a long time, cannot be completely explained by a glucocorticoid effect.

The phenomenon of adrenal cortex atrophy and decline in serum corticosterone levels caused by chronic administration of glucocorticoids has been previously reported (Donner et al., 2012). The process of adrenocortical hyperplasia induced by CRS may appear early in restraint stress and is related to the secretion of adrenocorticotropic hormone (ACTH) stimulated by corticotropin releasing factor (CRF) (Ulrich-Lai et al., 2006; Stanford, 2013). In this study, the serum corticosterone level dropped after the 3rd cycle of CRS, indicating activation of HPA axis during CRS is not continuous. The adrenal cortex remains proliferative for some time after the end of the restraint. The reason for this phenomenon may be that the recovery of the adrenal gland structure may take longer, or that in the central nervous system the CRF – ACTH regulatory pathway is still exerting its effect.

**FIGURE 11:**
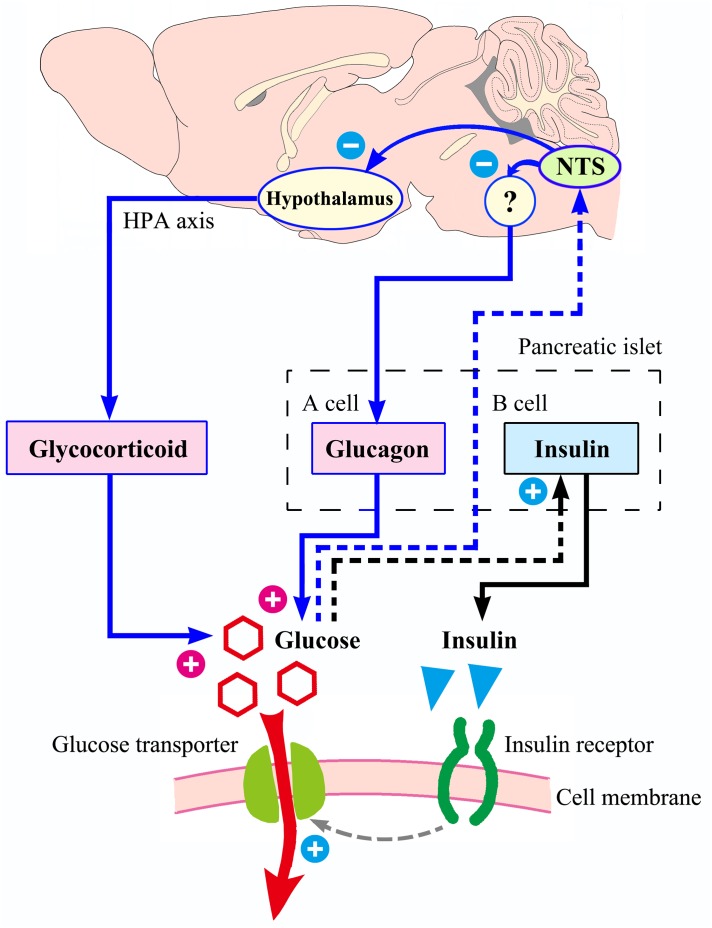
Possible process of NTS participating in blood glucose homeostasis. With increased blood sugar, the ability of islet beta cells to secrete insulin is enhanced. Insulin acts on its receptors to increase the efficiency of glucose transport into the cell, and blood glucose level decrease. The above mechanism (shown in black) does not involve the central nervous system. The regulation of blood glucose homeostasis by NTS is presented in blue. If the NTS feels that the blood glucose concentration rises, it may reduce the intensity of glucagon secretion by the islet alpha cells through the parasympathetic neural circuit (not completely clear, with question marks), and it can also send signals to the hypothalamus, reducing the excitability of the HPA axis and the release of glucocorticoids. Therefore, NTS may be the hub of a larger negative feedback regulation loop that compensates for the small loop of insulin regulation. If the NTS is damaged by the CRS, the insulin regulation mechanism is still intact, but the ability of regulation is limited.

From an anatomical point of view, the NTS is a special visceral sensory nucleus responsible for the transmission of sensory signals to the brain regions such as dorsal motor nucleus of the vagus, nucleus ambiguous, parabrachial nucleus, paraventricular nucleus of hypothalamus, to participate in the regulation of blood pressure, taste, respiration, visceral activity and stress responses (Rinaman, 2010; Alheid et al., 2011; Cutsforth-Gregory and Benarroch, 2017; Myers et al., 2017). Studies have confirmed that NTS has a distribution of blood glucose sensitive neurons (Oomura and Yoshimatsu, 1984; Yettefti et al., 1995; Dallaporta et al., 1999; Himmi et al., 2001), but the exact location of these neurons in the mouse NTS has not been reported in detail and cannot guide our experimental procedures. We first observed the distribution of apoptotic neurons caused by CRS, defined the brain regions of CRS lesions, and then observed which of these brain regions were excited by glucose. Through this screening, acNTS was confirmed as a key site. This region is bilaterally distributed, located in the middle of the Y-shaped NTS, just in front of the confluence zone and belongs to the cephalic part of the commissural subnucleus (McRitchie and Tork, 1993, for humans; Ganchrow et al., 2014, for C57BL/6J mice). The surgery of acNTS injury was only performed on the right side (after bilateral lesions, blood glucose elevation was far greater than unilateral, but the mice were in poor condition, which was not conducive to long-term experiments), and fasting blood glucose was observed to increase gradually after surgery. The process of NTS injury leading to elevated blood glucose levels can be explained using Figure 11. Peripheral tissue has a negative feedback regulation loop on blood glucose homeostasis. After the increase of blood glucose concentration, islet beta cells secrete more insulin. Under the synergistic action of insulin receptor signaling and glucose transporters (Chen et al., 2015), the cells accelerate the uptake of glucose and reduce the blood glucose level. However, if glucose continues to be produced and beta cells become overwhelmed, neuromodulation is necessary. It has been reported that the brain is involved in the regulation of food intake, sugar metabolism, and energy balance (Schwartz et al., 2000, 2013; Deem et al., 2017; Hackett and Steptoe, 2017). According to previous literature, NTS sends information to other parts of the central nervous system to regulate glucagon secretion by affecting the parasympathetic and sympathetic systems (Osundiji and Evans, 2013). This mechanism can prevent the breakdown of glycogen and reduce the rate of glucose production, thereby lowering blood glucose, and constitute another negative feedback regulation loop with brain involvement. NTS projects to the magnocellular divisions of medial periventricular hypothalamus (Herman, 2018) to regulate the activation of the HPA axis and reduce the release of glucocorticoids. This pathway also has the effect of lowering blood glucose levels. If the NTS is damaged and the blood glucose level cannot be sensed in time, these regulatory mechanisms will not work, and glucose will continue to be produced and accumulate in the blood. Intensive insulin secretion and islet beta cell proliferation can provide a temporary buffer, but over time, blood glucose will eventually break the limit and develop into hyperglycemia.

Chronic restraint stress causes hyperglycemia in about 1/3 but not all individuals in both mice and rats (Liang et al., 2013). However, after acNTS mechanical injury, all mice showed hyperglycemia, indicating that the body has a certain resistance or protective mechanism against the neural injury caused by CRS, and there are individual differences in the way of protection intensity or the protective effect. Stress hormones causing impairment to neurons (Arnsten, 2009; Popoli et al., 2012; Timmermans et al., 2013; Sousa, 2016) is the most likely factor. The corticosterone levels in the 1st and 2nd cycle of CRS were significantly higher than those in the non-restraint control, whereas neuronal apoptotic injury was already present in NTS at the end of the 1st cycle (Supplementary Figure S5). Neuronal apoptosis still exists at the end of the 4th cycle, which is a continuation of this injury process. Studies have shown that individuals with more elevated levels of corticosteroids in the early stage of stress are less affected later during chronic stress (Kim et al., 2013). This difference in response to stress stimuli may be an important cause of differential neuronal damage in chronic stress, but the exact mechanism is not yet clear. As there was no time-course investigation of acNTS injury, the effects of corticosterone induced neuronal injury and the relationship between neuronal damage and hyperglycemia deserve further study. On the other hand, neural secretions such as brain derived neurotrophic factor (BDNF) may provide protective effects against neuron injury (Zhao et al., 2017). In the absence of physical exercise, the secretion of BDNF is reduced, and its protective effect on neurons is reduced (Vaynman and Gomez-Pinilla, 2006). We have observed a significant decrease in BDNF expression in the cerebral cortex during chronic stress (Li et al., 2012). However, all CRS individuals show this change, suggesting a lack of neurotrophic factors are not a direct cause. The mechanism of protection and tolerance of the neural injury effects of CRS requires further study.

The experimental methods of this study have some special details. First, the binding strength of 6 h per day is based on previous experiments (Liang et al., 2013). If the restraint strength is weaker than 4 h/day, rodents do not have symptoms of hyperglycemia; if the restraint is stronger than 8 h/day, the animal’s health is adversely affected. The restraint period was selected from 0:00 to 6:00 a.m. every day, so that it contains a peak period of mouse activity (Jud et al., 2005). If the restraint operation is performed during the day, the effect on the exercise restriction is not as strong as the night restraint. Since the mouse sleeps most of the day, the daytime restraint may additionally cause disturbances in sleep. The room temperature should be maintained at 16–18°C during restraint, allowing the body temperature of mice to remain normal. The restraint device in this study caused some interference with heat dissipation on the body surface of mice, so the possible stress from abnormal body temperature regulation may be a factor, and its impact needs further analysis. It was reported that simple restraint stress does not activate the HPA axis (Flak et al., 2012). In our experiments, early activation of the HPA axis in CRS was observed, which may be related to interference with body temperature regulation. Second, in each cycle, the mice were restrained for 7 days and then free to live for 3 days before testing. The purpose of this arrangement is to both restore the mice properly and eliminate acute phase reactions caused by stress. In mice that have never been restrained, blood glucose and corticosterone levels return to resting levels within 2 h after 15 min of binding (Supplementary Figure S3). In addition, the interval between GTT and ITT was 3 days. Therefore, it is sufficient to set aside a recovery time of 3 days after the restraint. Third, the four cycles of restraint ensure that the mice’s fasting hyperglycemic state is maintained for at least 3 weeks (the longest period of the previous experiment, see Supplementary Figure S2; the animals were then sacrificed and used for experimental testing). Increasing the number of restraint cycles does not produce new individuals with hyperglycemia. No individuals with hypoglycemia was observed during CRS. Fourth, in this study, neurons of acNTS had characteristics of apoptosis, such as cell concentration, Caspase-3 expression, and TUNEL positive staining without inflammation. These results suggest that the neurons had severe injury, but to confirm that this injury was apoptosis, further testing is still needed. One reason is that the TUNEL assay does not strictly distinguish between necrosis, autolysis and apoptosis (Charriaut-Marlangue et al., 1995; Grasl-Kraupp et al., 1995). The primary objective of this study was to observe the location of neuronal injury in the brain under conditions of CRS. At present, more methods are needed to detect apoptosis for the *in situ* detection.

## Conclusion

Our study revealed that injury (likely apoptosis) of blood glucose sensitive neurons in acNTS plays an important role in CRS-induced hyperglycemia in mice. CRS impairs glucose-sensitive neurons of acNTS and thus leads to insulin-resistant hyperglycemia. The CRS model has potential value in the study of the pathological mechanism of stress-induced hyperglycemia. Further research is needed to determine how acNTS participates in the regulation of blood glucose homeostasis and the protective mechanisms of CRS-related brain injury.

## Author Contributions

XiZ and WB conceived and designed the study. XiZ, WB, JW, and XL performed the experiments. XiZ wrote the first draft of the manuscript. WB and XuZ revised and improved the manuscript. GY and JZ reviewed the manuscript.

## Conflict of Interest Statement

The authors declare that the research was conducted in the absence of any commercial or financial relationships that could be construed as a potential conflict of interest.
