# Supplementary material for: Hyperglycemia Induced by Chronic Restraint Stress in Mice Is Associated With Nucleus Tractus Solitarius Injury and Not Just the Direct Effect of Glucocorticoids
Source: Front Neurosci. 2018 Dec 19;12:983. doi: 10.3389/fnins.2018.00983 (PMC6305899; doi:10.3389/fnins.2018.00983)
Supplement: Supplementary file 1 [file Data_Sheet_1.DOCX]

**Supplementary material**

**Part 1 Mouse restraint operation**

In this study, the basic method of mouse restraint referred to Bowers et al., 2008, and was modified by using a 50 ml plastic centrifuge tube (Guo et al., 2017). First, a 4 mm diameter hole was drilled in the bottom of the centrifuge tube to allow the mouse to breathe. Then 4 holes (4 mm in diameter) were made at equal distances around the neck of the tube to allow urine to flow out. Finally, a hole of the same diameter was drilled in the center of the lid. The tail of the mouse under restraint was transmitted through the hole.

Place the mouth of the restrained tube close to the mouse's head, and gently push the mouse into the tube. A few mice need to make multiple attempts at the beginning. In the restrained tube, there was still some space around the body of the mouse, but it was not enough to turn around. During the beginning 1 to 2 periods of restraint, the mouse's forelimb kept digging for several minutes, and this behavior recurred frequently, but after 3 days, mice under restraint apparently calmed down. The restrained tubes were placed on a bedding made of aspen shavings (the bottom of each tube was higher than the mouth to avoid fecal contamination of the mouse body), maintaining the room temperature at 16 – 18 ℃. Our previous experiments found that a slightly lower room temperature ensures that the body temperature of the restrained mice remains normal.

After 6 hours of restraint, the lid was opened and the mice withdrew from the tube on their own. Control mice and CRS mice entered the restraint devices at the same time, but then the control mice were released (no more than 1 min) and free to move. Restrained tubes and lids were collected and cleaned immediately, then dried for reuse.

**Part 2 Supplementary figures**


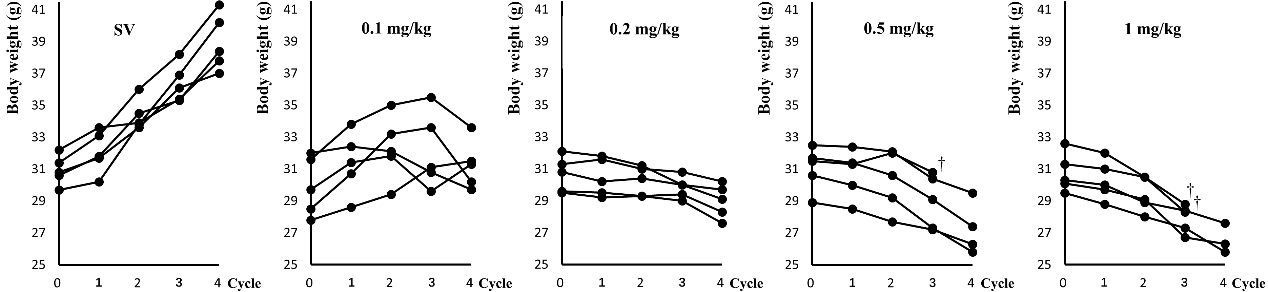


**FIGURE S1** | Experiments of DEX injection concentration. The effect of 0.1 mg/kg DEX on body weight was quite variable, and previous reports also believed that this concentration was not sufficient to cause typical insulin resistance. At a concentration of 0.2 mg/kg, the body weight of the mice was slowly reduced, and their activities and health were normal. Concentrations above 0.5 mg/kg can cause mice to become apathetic and even die, suggesting that the effects are too strong. ^†^ The mouse died. The mice used in this experiment were different from those in the results section.


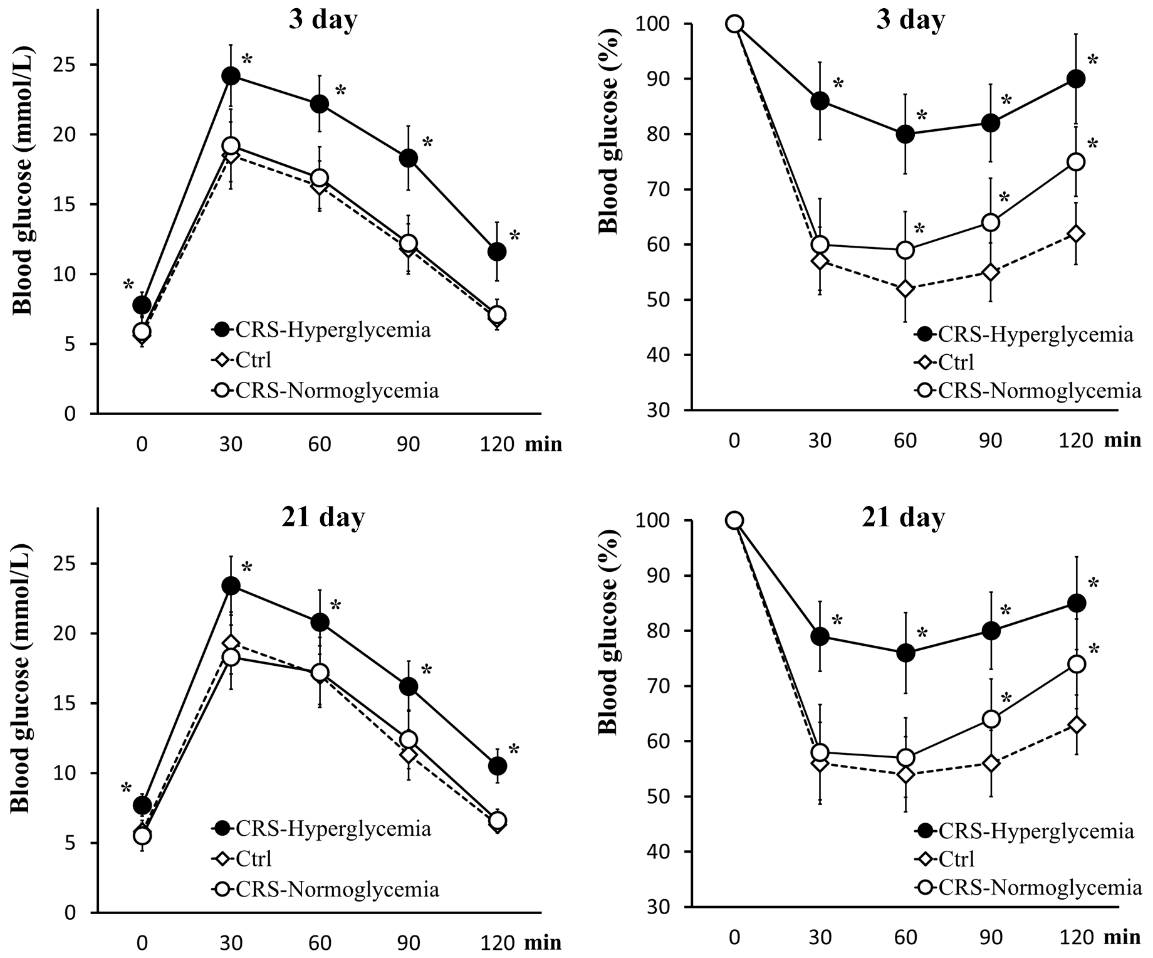


**FIGURE S2** | The pre-experimental results of the GTT and ITT on the 3rd and 21st day after CRS. On day 3 and day 21 after CRS, insulin-resistant hyperglycemia persisted in mice. The operation of this experiment is the same as the “Material and Methods” of the text, and mice used in CRS-hyperglycemia (N = 6), CRS-normoglycemia (N = 14), and control (Ctrl, N = 6) group were different from those in the results section. Data were shown as means ± SEM, ^*^ *p* ＜ 0.01.


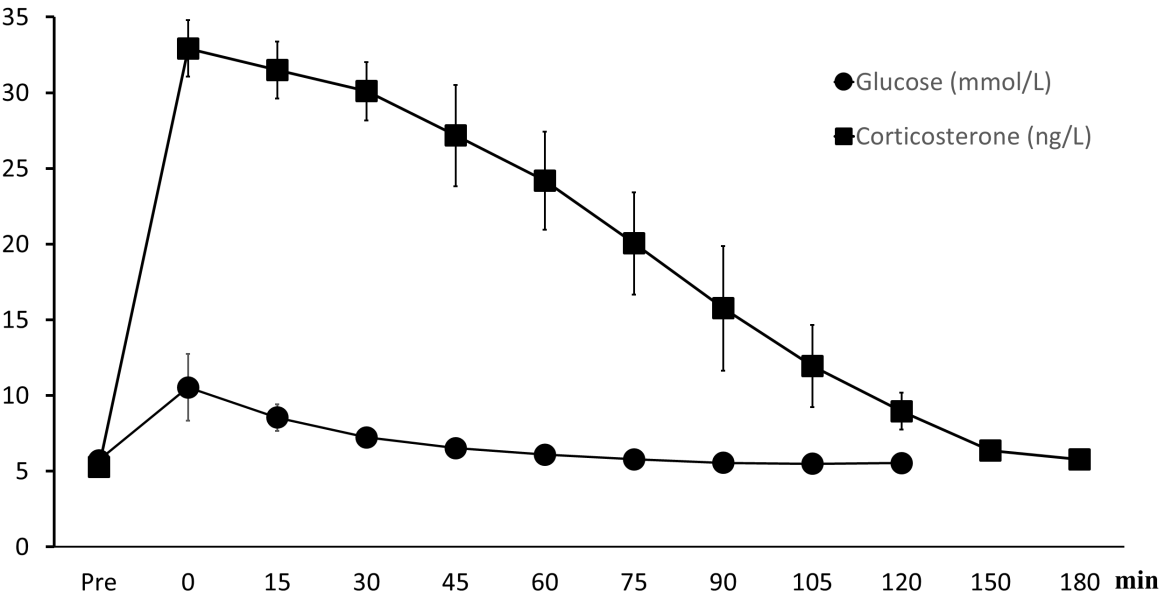


**FIGURE S3** | The effect of acute restraint on blood glucose and serum corticosterone levels. Previously unrestraint mice (N = 6) were fasted for 4 h, restrained for 15 min and then free to move. Changes in blood glucose and serum corticosterone levels within 2–3 hours were monitored. The blood glucose concentration approximately doubled during restraint and completely returned to fasting levels within 2 hours after restraint. Serum corticosterone increased significantly to 6 times normal level during restraint and returned to resting levels after 3 hours of release. In this study, time intervals between the experimental tests and the restraint operation were much longer than 3 hours, so the acute effects of the restraint could be eliminated.


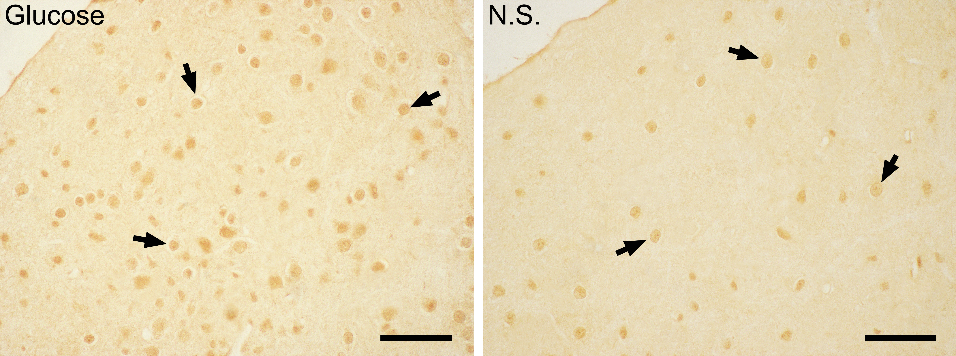


**FIGURE S4** | Expression of c-Fos in mouse acNTS at 1.5 h after glucose injection. Compared with saline injected mice, c-Fos positive nuclei of acNTS in mice injected with glucose significantly increased. Micrographs were taken from the coronal section interaural －3.76 mm, bregma －7.56 mm. N.S., Normal saline; **↑** Neurons with c-Fos positive nuclei. Bar = 100 μm.


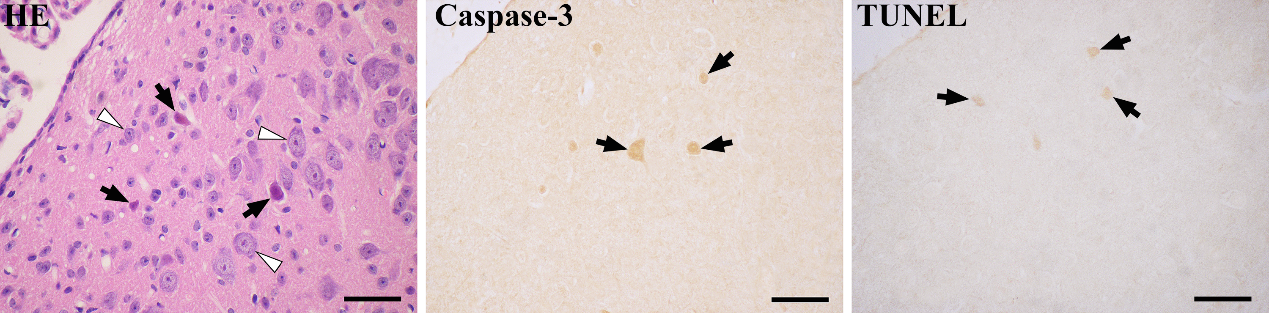


**FIGURE S5** | Observation of apoptotic neurons in acNTS at the end of the 1st cycle of restraint. At the end of the 1st cycle of restraint, only a few mice (at most 1 in 6) exhibited neuronal apoptotic injury in the NTS. Whether this mouse eventually develops insulin-resistant hyperglycemia and whether NTS neurons continue to undergo apoptosis at the end of the 4th cycle remains to be further studied. The mice used in this experiment were different from those in the results section. Micrographs were taken from the coronal section interaural －3.76 mm, bregma －7.56 mm. ∆ Normal neurons; **↑** Apoptotic neurons. Bar = 100 μm.
